# Real-world treatment patterns and clinical outcomes in patients with AML from 65 to 74 years unfit for first-line intensive chemotherapy in Japan

**DOI:** 10.1007/s12185-026-04193-3

**Published:** 2026-03-19

**Authors:** Fumiaki Fujii, Masahiro Onozawa, Shota Yoshida, Naoki Miyashita, Daisuke Hidaka, Reiki Ogasawara, Mutsumi Takahata, Junichi Hashiguchi, Shota Yokoyama, Masahiro Chiba, Tomoyuki Saga, Taku Shimizu, Ikumi Kasahara, Akio Shigematsu, Katsuya Fujimoto, Satoshi Iyama, Tetsuyuki Igarashi, Shinichi Ito, Yoshihito Haseyama, Mizuha Kosugi-Kanaya, Takeshi Kondo, Takanori Teshima

**Affiliations:** 1 Department of Hematology, Faculty of Medicine, Graduate School of Medicine, Hokkaido University, Kita 15, Nishi 7, Kita-Ku, Sapporo, 0608638 Japan; 2 Department of Hematology, Sapporo Hokuyu Hospital, Sapporo, Japan; 3 Department of Hematology, Sapporo Kosei General Hospital, Sapporo, Japan; 4 Department of Internal Medicine/General Medicine, Kitami Red Cross Hospital, Kitami, Japan; 5 Department of Hematology, Obihiro Kosei Hospital, Obihiro, Japan; 6 Department of Hematology, Asahikawa City Hospital, Asahikawa, Japan; 7 Department of Hematology, Kin-Ikyo Chuo Hospital, Sapporo, Japan; 8 Department of Hematology, Teine Keijinkai Hospital, Sapporo, Japan; 9 Department of Hematology, Sapporo City General Hospital, Sapporo, Japan; 10 Department of Hematology, Kushiro Rosai Hospital, Kushiro, Japan; 11 Department of Hematology, NHO Hokkaido Cancer Center, Sapporo, Japan; 12 Department of Hematology, Sapporo Medical University School of Medicine, Sapporo, Japan; 13 Department of Hematology, Tenshi Hospital, Sapporo, Japan; 14 Department of Hematology, Hakodate Municipal Hospital, Hakodate, Japan; 15 Department of Hematology, Tonan Hospital, Sapporo, Japan; 16 AbbVie GK, Tokyo, Japan; 17 Blood Disorders Center, Aiiku Hospital, Sapporo, Japan

**Keywords:** Venetoclax, Azacitidine, Elderly patients, Treatment patterns, Acute myeloid leukemia

## Abstract

Venetoclax (VEN), a BCL-2 inhibitor, was approved in Japan in March 2021, for acute myeloid leukemia (AML). We retrospectively analyzed the impact of VEN approval on treatment patterns and outcomes in older AML patients aged 65–74 years unfit for intensive chemotherapy in Japan. Using the Hokkaido Leukemia Net database, we categorized 101 patients into pre-VEN (*n* = 46) and post-VEN (*n* = 55) cohorts, excluding those who had acute promyelocytic leukemia or received intensive chemotherapy. Following VEN approval, VEN + azacitidine (AZA) became the most frequently used initial regimen (56%). Despite higher rates of *TP53* mutations and complex karyotypes (35.5%), VEN + AZA achieved comparable response rates (CR + CRi: 64.5%) and overall survival (OS, median 11.7 months) to r7 + 3 (CR + CRi: 64.5%, median OS: 13.1 months), and superior outcomes to cytarabine + aclarubicin + G-CSF (CAG, CR + CRi 37.5%, median OS 6.8 months) or AZA monotherapy (CR + CRi 12.5%, median OS 4.5 months). Early mortality at 60 days from diagnosis was lower with VEN + AZA (3.2%) than with reduced-dose cytarabine plus anthracycline (r7 + 3) (12.9%), CAG (26.7%), or AZA monotherapy (18.8%). Our findings demonstrate a substantial shift in real-world treatment practices following VEN approval and suggest that VEN + AZA is an effective option for older AML patients with adverse genetic features.

## Introduction

Acute myeloid leukemia (AML) is a clonal hematopoietic malignancy characterized by high molecular and pathogenic heterogeneity [1, 2]. AML primarily affects older adults, and the median age at diagnosis is 69 years, and AML is most frequently diagnosed in individuals aged 65–74 years [3]. In Japan, the annual incidence of AML is 5.4 per 100,000 individuals, and this rises to 10–17 per 100,000 for those older than age 69 years [4]. The standard of care treatment for young and fit patients with AML is intensive chemotherapy (IC), often followed by hematopoietic cell transplantation (HCT), while more than half of elderly patients with age 65 or more are not candidates for IC or HCT. Venetoclax (VEN), a BCL-2 inhibitor, has expanded the available options for patients with AML ineligible for intensive chemotherapy. VEN was approved in Japan since March 2021. The international guidelines recommend VEN combinations as a preferred regimen for the treatment of patients with AML who are ineligible for IC due to age and/or comorbidities [5]. Limited data are available on real-world outcomes with VEN regimen comparing to other lower intensity therapies in this patient population, because superiority of VEN + Azacitidine (AZA) was shown by the comparison to AZA monotherapy as a control [6, 7]. The development of optimal treatment algorithms for older patients is needed.

This study aimed to evaluate the impact of VEN approval on treatment selection and outcomes among elderly AML patients ineligible for IC, using real-world data before and after its introduction in Japan.

## Materials and methods

### Patients

**Fig. 1:**
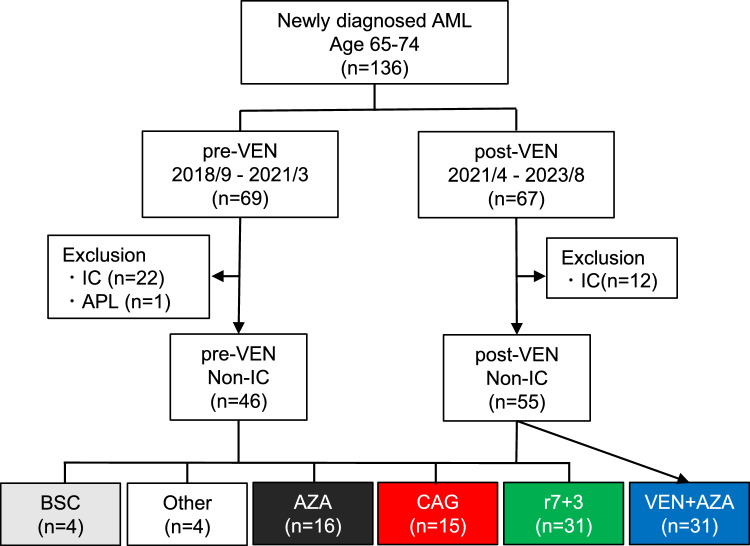
The consort graph of patients enrollment. AML, acute myelogenous leukemia; APL, acute promyelocytic leukemia; CAG, cytarabine + aclarubicin + granulocyte colony-stimulating factor; AZA, azacitidine; VEN, venetoclax; IC, intensive chemotherapy; r7 + 3, reduced-dose 7 + 3

Hokkaido Leukemia Net (HLN) is a regional prospective cohort study registering cases of newly diagnosed AML in Hokkaido, Japan (UMIN: 000048611). We retrospectively analyzed AML cases except for acute promyelocytic leukemia or intensive chemotherapy with age 65–75 years, which were registered in HLN between September 2018 and August 2023. Since VEN was approved in Japan in Mar 2021, we defined Sep 2018–Mar 2021 as “pre-VEN era” and Apr 2021–Aug 2023 as “post-VEN era.” The consort graph is shown in Fig. 1. This study was conducted in accordance with the Helsinki Declaration and was approved by the Hokkaido University Hospital institutional review boards (#015–0344). This exploratory study, HLN GML2024, was conducted as a subgroup study of HLN and was approved by the Hokkaido University Hospital institutional review boards (#023–0305). This study aims to assess clinical outcomes and treatment courses among unfit AML patients aged 65–74, analyzing patient-specific variables and disease profiles to identify factors influencing treatment efficacy.

Ineligibility for intensive chemotherapy was defined based on the treating physician’s assessment of patients’ age, performance status, comorbidities, and institutional practice. Only 7 days of standard-dose cytarabine (AraC, 100 mg/m^2^) and 3 days of idarubicin (IDA, 12 mg/m^2^) or 5 days of daunorubicin (DNR, 50 mg/m^2^) were defined as intensive chemotherapy. Any modification of dose or duration of the IDA + AraC or DNR + AraC regimen was defined as a reduced-dose 7 + 3 (r7 + 3).

### Analysis of genetic alterations

We developed a compact AML panel covering mutation hot spot of 16 genes including *TP53, CEBPA, NPM1, FLT3, KIT, NRAS, KRAS, CBL, PTPN11, DNMT3A, IDH1, IDH2, RUNX1*, *ASXL1*, *BCOR* and *SF3B1*. Multiplex polymerase chain reaction (PCR) products of genomic DNA were deep-sequenced at Research Institute for Microbial Disease, Osaka University (Osaka, Japan) using Miseq (Illumina, San Diego, CA). Only variants occurring with a variant allele frequency (VAF) of more than 10% were defined as mutations.

### Statistical considerations

The primary objective is describing the treatment pattern of the initial treatment and following treatment up to third cycle of treatment. The final data cutoff was June 30, 2024. The secondary objectives are describing treatment outcomes, including overall survival (OS), response rates (complete remission [CR] + CR with incomplete hematologic recovery [CRi]), event-free survival (EFS) and early mortality of 60 days after diagnosis and cause of death. Event-free survival (EFS) was defined as the time from diagnosis to primary induction failure, relapse or death from any cause, whichever occurred first. Primary induction failure was defined as failure to achieve complete remission (CR) after two cycles of treatment or a change in treatment regimen before achieving CR, whichever occurred first.

Continuous variables are described with median and ranges. Fisher’s exact test was used to compare categorical values and the Mann–Whitney U test was used to compare continuous values. OS was measured from the date of diagnosis until the date of death from any cause using the log rank test. Statistical significance was defined as a two-tailed *P* value < 0.05. All statistical analyses were performed with EZR ver 1.52 (Jichi Medical University Saitama Medical Center), which is a graphical user interface for R (The R Foundation for Statistical Computing, Vienna, Austria) [8].

## Results

A total of 136 AML patients aged 65–74 were identified from the Hokkaido Leukemia Net database. Excluding patients who received intensive chemotherapy (IC) and those with acute promyelocytic leukemia, 46 and 55 patients were registered in the pre-VEN and post-VEN groups, respectively (Fig. 1). In the pre-VEN era, 22 patients (31.9%) received IC, whereas in the post-VEN era, this number decreased to 12 patients (17.9%). Patients’ characteristics are shown in Table 1. Background characteristics of pre- and post-VEN era had comparable age distribution (median age 70 vs. 71), sex (M/F: 30/16 vs. 32/23), median follow-up days (320, range 11–1962 vs. 356, range 4–1188), *FLT3-ITD* (6/46 vs. 7/55), *TP53* mutation (8/46 vs. 14/55), complex karyotype (CK) (9/46 vs. 14/55), and European LeukemiaNet 2017 risk group (favorable/intermediate/adverse: 8/14/24 vs. 8/14/33) (Table 1).

**Table 1:** Patients’ characteristics of pre- and post-VEN era

	pre VEN *N* = 46	post VEN *N* = 55	*P*-value
Median age at diagnosis (range)	70 (65–74)	71 (65–74)	0.45
Male/Female	30/16	32/23	0.54
Median follow-up days (range)	320 (11–1962)	356 (5–1188)	0.10
WHO classifiction (2017)			
AML with recurrent genetic abnormalities, *n* (%)	5 (10.9)	0 (0.0)	0.017*
AML with MRC, *n* (%)	21 (45.7)	25 (45.5)	1.00
Therapy-related myeloid neoplasms, *n* (%)	2 (4.3)	4 (7.3)	0.69
AML, NOS, *n* (%)	18 (39.1)	26 (47.3)	0.43
M4 or M5, *n* (%)	12 (26.1)	11 (20.0)	0.49
*FLT3-ITD*, *n* (%)	6 (13.0)	7 (12.7)	0.77
*TP53* mutation, *n* (%)	8 (17.4)	14 (25.5)	0.35
Complex karyotype, *n* (%)	9 (19.6)	14 (25.5)	0.63
Complications at diagnosis, *n* (%)	27 (58.7)	26 (47.3)	0.25
Congestive heart failure/Condition requiring oxygen/Liver dysfunction/Renal dysfunction/Infection, *n* (%)	5/5/4/5/8	3/6/0/7/10	
ELN 2017 risk group			
Favorable, *n* (%)	8 (17.4)	8 (14.5)	0.91
Intermediate, *n* (%)	14 (30.4)	14 (25.5)	0.74
Adverse, *n* (%)	24 (52.2)	33 (60.0)	0.92

VEN venetoclax, AML acute myeloid leukemia, MRC myelodisplasia-related change, NOS not otherwise specified, ELN European LeukemiaNet.

**Fig. 2:**
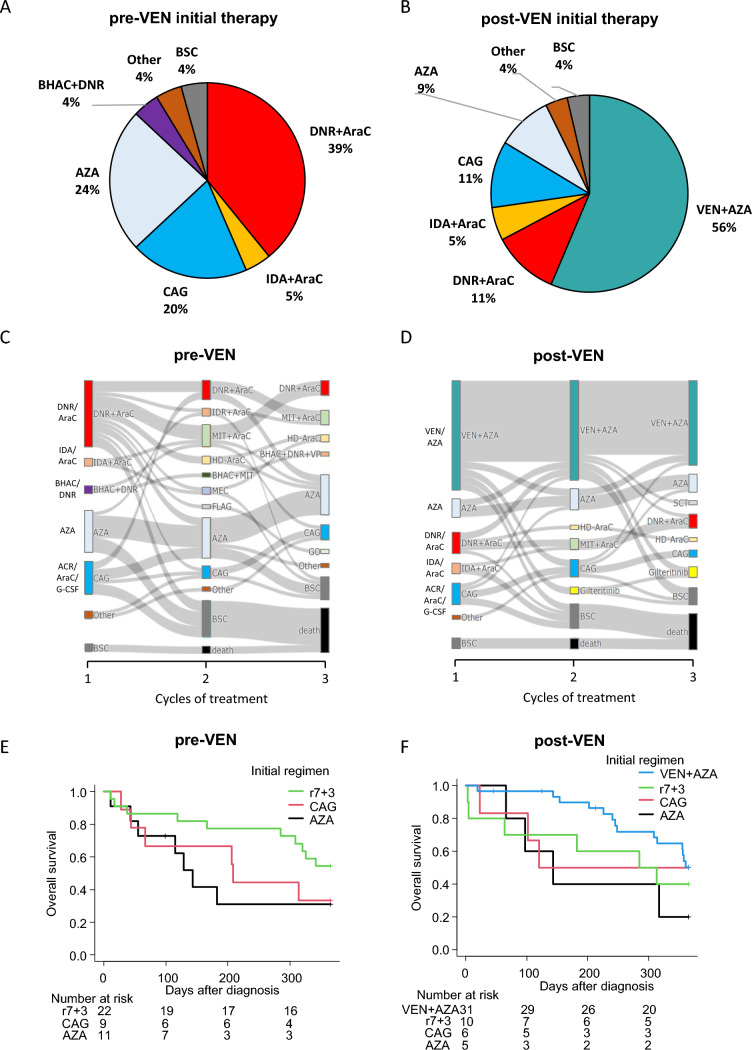
Distribution of initial therapy in pre-VEN **A** and post-VEN **B**. Treatment patterns over three cycles in the pre-VEN era **C** and in the post-VEN era **D**. Overall survival of patients treated by each initial regimen in pre-VEN **E** and post-VEN **F**. DNR: daunorubicin, AraC: cytarabine, HD-AraC: high dose cytarabine, IDA: idarubicin, BHAC, behenoyl AraC; MIT, mitoxantrone; CAG, cytarabine + aclarubicin + granulocyte colony-stimulating factor; AZA, azacitidine; VEN, venetoclax; BSC, best supportive care; GO, gemtzumab ozogamicin; SCT, stem cell transplantation

In pre-VEN era, most frequently used initial regimen was reduced-dose 7 + 3 (r7 + 3; idarubicin or daunorubicin + cytarabine; 44%), following AZA (24%) and CAG (low-dose cytarabine, aclarubicin hydrochloride and granulocyte colony-stimulating factor) (20%) (Fig. 2A). In post-VEN era, more than half of the patients were initially treated by VEN + AZA (56%) following r7 + 3 (16%) and CAG (11%), AZA (9%) (Fig. 2B). The Sankey plot illustrated the initial treatment distribution and the following treatment regimen before and after VEN approval (Fig. 2C, D). In the pre-VEN era, AZA is the most frequent third cycle treatment (*n* = 11, 32.4%), following best supportive care (BSC) defined as palliative treatment aimed at symptom control, including antibiotics and transfusion, without cytotoxic therapy (*n* = 6, 17.6%). In the post-VEN era, VEN + AZA was remained the most frequently used regimen at the third cycle treatment (*n* = 24, 45.3%), and AZA (*n* = 5, 9.4%) was the second. The median number of treatment cycles (range) was three (1–22) in the VEN + AZA group, two (1–5) in the r7 + 3 group, one (1–4) in the CAG group and two (1–24) in the AZA monotherapy group. Overall survivals of four major initial regimens (VEN + AZA, r7 + 3, CAG, and AZA) were shown for pre- and post-VEN era (Fig. 2E, F). Across both the pre-VEN and post-VEN eras, hematopoietic stem cell transplantation was performed in four patients (4/31, 12.9%) in the VEN + AZA group, two patients (2/31, 6.5%) in the r7 + 3 group, none (0/15, 0.0%) in the CAG group and one patient (1/16, 6.3%) in the AZA group.

**Fig. 3:**
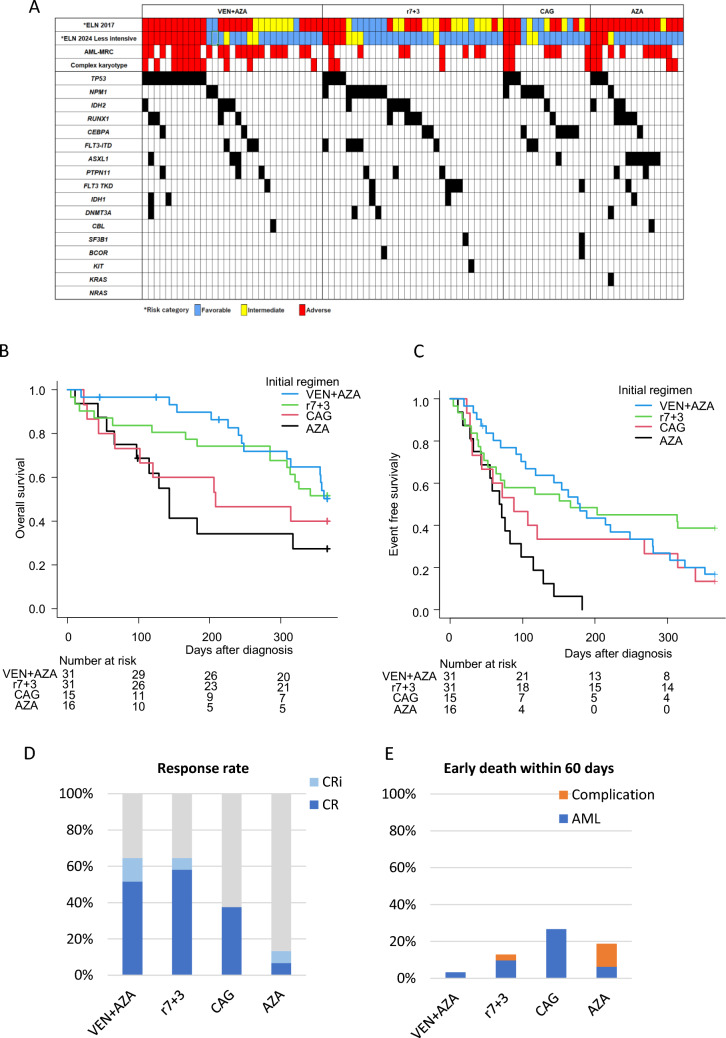
**A** The genetic landscape of the patients in each treatment group. **B** Overall survival of patients treated with each initial regimen. Pre- and post-VEN cohort were combined for r7 + 3, CAG, AZA group. **C** Event-free survival of patients treated with each initial regimen. Pre- and post-VEN cohort were combined for r7 + 3, CAG, AZA group. **D** CR and CRi rates of patients treated with each initial regimen. **E** Early death within 60 days of patients treated with each initial regimen. VEN, venetoclax; AZA, azacitidine; r7 + 3, reduced-dose cytarabine plus anthracycline; CAG, cytarabine + aclarubicin + granulocyte colony-stimulating factor

Genetic analysis of cases in each treatment group is shown in Fig. 3A. Case, initially treated by VEN + AZA group, had a higher frequency of *TP53* mutations (11/31, 36.7%) compared to other regimen group r7 + 3 (4/31, 12.9%), CAG (3/15, 20.0%), AZA (3/16, 18.8%), suggesting a preferential selection of this regimen for high-risk patients. Overall survival and event-free survival of the pre- and post- VEN combined cohorts of patients treated with r7 + 3, CAG and AZA were compared to the VEN + AZA group (Fig. 3B, C). Median OS of VEN + AZA, r7 + 3, CAG, and AZA was 11.7, 13.1, 6.8 and 4.5 months, respectively (Log-rank, *p* = 0.0784). Median EFS of VEN + AZA, r7 + 3, CAG, and AZA was 5.8, 5.5, 2.9 and 2.3 months, respectively (Log-rank, *p* = 0.001). Patients’ characteristics of each initial treatment regimen are shown in Table 2. Representing higher prevalence of CK and *TP53* mutation, VEN + AZA group showed higher number of adverse risk patients both in ELN 2017 (21/31, 67.7%) and ELN 2024 less intensive (11/31, 35.5%) risk group. CR + CRi rates were comparable in VEN + AZA (64.5%) and r7 + 3 (64.5%), which is higher than in CAG (37.5%) and AZA (12.5%) (Fig. 3D). Early mortality rate at 60 days from diagnosis was lower in VEA + AZA (3.2%), compared to r7 + 3 (12.9%), CAG (26.7%), or AZA monotherapy (18.8%) (Fig. 3E). Despite the higher number of patients with CK and *TP53* mutation among VEN + AZA group, OS and response rates were comparable to those of r7 + 3.

**Table 2:** Patients’ characteristics of each initial treatment regimen

	VEN + AZA N = 31	r7 + 3 N = 31	CAG N = 15	AZA N = 16	*P*-value
Median age at diagnosis (range)	71 (65–74)	70 (66–74)	71 (66–74)	70 (65–74)	0.86
Male/Female	15/16	20/11	11/4	10/6	0.37
Median WBC at diagnosis (range)/μL	2,500 (760–188,510)	13,700 (860–461,400)	18,000 (1930–215,790)	2130 (400–153,900)	0.09
Median number of treetment (range)	3 (1–22)	2 (1–5)	1 (1–4)	2 (1–24)	
AML classification-WHO 2017, *n* (%)					0.15
AML with recurrent genetic abnormalities	0 (0.0)	2 (6.5)	0 (0.0)	0 (0.0)	
AML with MRC	15 (48.4)	8 (25.8)	6 (40.0)	1 (6.3)	
Therapy-related myeloid neoplasms	3 (9.7)	2 (6.5)	0 (0)	1 (6.3)	
AML, NOS (none M4, M5)	9 (29.0)	16 (51.6)	7 (46.7)	12 (75.0)	
AML, NOS (M4 or M5)	4 (12.9)	3 (9.7)	2 (13.3)	2 (12.5)	
Complex karyotype, *n* (%)	11 (35.5)	3 (9.7)	3 (20.0)	4 (25.0)	0.11
*TP53* mutation, *n* (%)	11 (35.5)	4 (12.9)	3 (20.0)	3 (18.8)	0.19
ELN 2017 risk category, *n* (%)					0.04
Favorable	3 (9.7)	7 (22.6)	4 (26.7)	0 (0.0)	
Intermediate	7 (22.6)	13 (41.9)	4 (26.7)	1 (6.3)	
Adverse	21 (67.7)	11 (35.5)	7 (46.7)	15 (93.8)	
ELN 2024 Less intensive risk category, *n* (%)					0.36
Favorable	17 (54.8)	23 (74.2)	10 (66.7)	12 (75.0)	
Intermediate	3 (9.7)	4 (12.9)	2 (13.3)	1 (6.3)	
Adverse	11 (35.5)	4 (12.9)	3 (20.0)	3 (18.8)	
Complications at diagnosis, *n* (%)					0.78
Congestive heart failure	2 (6.5)	2 (6.5)	2 (13.3)	1 (6.3)	
Condition requiring oxygen	0 (0.0)	4 (12.9)	4 (26.7)	1 (6.3)	
Liver dysfunction	0 (0.0)	1 (3.2)	1 (6.7)	1 (6.3)	
Renal dysfunction	3 (9.7)	4 (12.9)	2 (13.3)	0 (0.0)	
Infection	4 (12.9)	7 (22.6)	4 (26.7)	1 (6.3)	
Number of complication, *n* (%)					0.97
1	3 (9.7)	9 (29.0)	3 (20.0)	2 (12.5)	
2	3 (9.7)	3 (9.7)	2 (13.3)	1 (6.3)	
≥ 3	0 (0.0)	1 (3.2)	2 (13.3)	0 (0.0)	

VEN venetoclax, AZA azacitidine, CAG acracinomycin + cytarabine + G-CSF, r7 + 3 reduced 7 + 3, WBC white blood cell, WHO World Health Organization, AML acute myeloid leukemia, MRC myelodisplasia-related change, NOS not otherwise specified, ELN European LeukemiaNet.

**Fig. 4:**
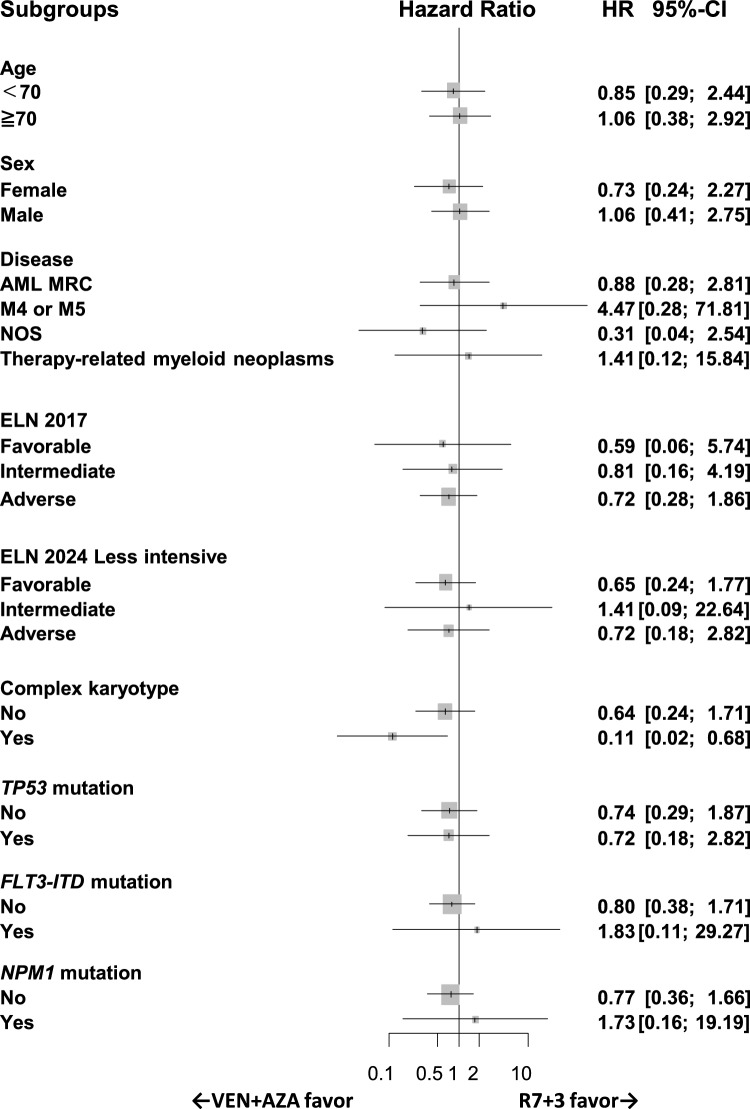
Forest plot showing subgroup analysis comparing survival between patients initially treated with VEN + AZA and those treated with r7 + 3. Hazard ratios (HR) with 95% confidence intervals (CI) are shown for each subgroup. An HR less than one indicates a survival benefit favoring VEN + AZA. Subgroups included cytogenetic and molecular risk factors, AML subtype, and baseline characteristics. VEN, venetoclax; AZA, azacitidine; r7 + 3, reduced-dose 7 + 3; CK, complex karyotype; ELN, European LeukemiaNet; MRC, myelodysplasia-related changes

Patients initially treated by VEN + AZA (*n* = 31) were compared to those who were treated by r7 + 3 (*n* = 31, pre- and post-VEN). Factors that favored 1 year OS for VEN + AZA vs r7 + 3 (Fig. 4). Forest plot showed that AML M4, 5 favored r7 + 3, whereas complex karyotype favored VEN + AZA.

## Discussion

Before the approval of VEN, treatment options for patients with AML who were ineligible for IC were limited to AZA, CAG or low-dose cytarabine. Reduced modification of the standard 7 + 3 regimen is also considered as an option for IC-ineligible patients. The clinical efficacy of VEN in combination with AZA was demonstrated in IC-ineligible AML patients, and its approval leads to a significant change in treatment selection. In line with this shift, the proportion of patients aged 65–74 years who actually received intensive chemotherapy decreased substantially after the approval of venetoclax, from 31.9% in the pre-VEN era to 17.9% in the post-VEN era. This observation suggests that, in real-world practice, a subset of patients who might previously have been considered eligible for intensive chemotherapy was instead treated with VEN + AZA following its introduction.

Several real-world studies have compared VEN-based regimens with the standard 7 + 3 regimen, often using propensity score matching due to significant differences in baseline characteristics between cohorts [9–11]. A retrospective analysis of electronic health record (EHR)-derived data focusing on patients aged 60–75 years in the USA demonstrated better survival in patients who received 7 + 3 compared to those who received VEN + hypomethylating agents (HMA) (22 vs. 10 months; HR, 0.53; 95% CI, 0.40–0.60) [10]. In this analysis, VEN + HMA patients were older and more likely to have secondary AML, adverse cytogenetics, and adverse mutations. Survival advantage of IC was reduced by half after propensity score matching. To ensure a direct and fair comparison, we focused on more specific age group (65–74 years) with comparable baseline characteristics. Additionally, data were collected before and after VEN approval in Japan to assess its impact while minimizing potential bias, particularly in selecting reduced-dose 7 + 3 regimens in the VEN era.

In the pre-VEN era, AZA monotherapy was the most frequently used regimen in the second- and third-line settings. The introduction of VEN resulted in a substantial shift in treatment strategies, with VEN + AZA becoming the most frequently used regimen from the first to the third cycle in the post-VEN era. Despite a higher prevalence of complex karyotype (CK) and *TP53* mutations in the VEN + AZA group, patients who initially received VEN + AZA demonstrated comparable response rate and survival to r7 + 3, and lower early mortality. In this study, despite genomic alterations were analyzed retrospectively, significantly higher number of *TP53* mutation were detected in VEN + AZA group. *TP53* mutation is a well-established poor prognostic factor in AML, regardless of whether patients receive intensive or reduced-intensity chemotherapy [5, 12–14]. In the post-VEN era, the proportion of AML with myelodysplasia-related changes (MRC) was 21 out of 31 (67.7%) in the VEN + AZA group and 2 out of 9 (22.2%) in the r7 + 3 group (Fisher’s exact test, *p* = 0.023). In this retrospective analysis, there appeared to be a tendency to select the VEN + AZA regimen for patients with AML MRC, which may explain the higher frequency of *TP53* mutations observed in the VEN + AZA group. Although VEN shows an initial response in patients with *TP53* mutations, these patients eventually relapse and have poor outcomes. Comprehensive genomic profiling (CGP) is just approved for various hematological malignancy in Japan. The reduced early mortality observed in the VEN + AZA group may allow clinicians more time to obtain genomic data before deciding on personalized treatment strategies. In the near future, molecularly targeted agents in combination with VEN could further improve survival outcomes in this patient population.

Survival outcomes of patients treated with r7 + 3 seemed largely consistent with that of patients undergone intensive therapy in previous real-world studies [15, 16]. On the other hand, BSC was selected for quarter to half of the elderly AML patients in real-world data in pre-VEN era [16–19]. Only 4% of the patients were chosen BSC option in our cohort. The introduction of hypomethylating agents and VEN has dramatically expanded treatment options for elderly AML.

Several studies have suggested that monocytic AML (FAB M4/M5) and TP53-mutated AML are associated with inferior responses to venetoclax-based regimens [20–22]. In our cohort, patients with FAB M4/M5 appeared to derive greater benefit from r7 + 3, whereas TP53 mutation did not clearly favor either regimen. One possible explanation is that monocytic AML is characterized by lower BCL-2 dependency and greater reliance on alternative anti-apoptotic proteins such as MCL-1, which may confer intrinsic resistance to venetoclax. In contrast, TP53-mutated AML often exhibits an initial response to VEN-based therapy but rapidly develops resistance, leading to poor long-term outcomes regardless of treatment intensity [22].

There are several limitations in this study. This study involved a small number of patients who met the age criteria, so a direct comparison with previous studies is difficult due to inter-study variability. The choice of VEN + AZA was not randomized, increasing the potential for patient selection bias. Given the retrospective nature and lack of propensity score matching, comparative results should be interpreted with caution. Despite these limitations, our study highlights a clear transition in AML treatment strategies following the introduction of VEN. The introduction of VEN has led to a significant change in the treatment approach for younger elderly AML patients who are ineligible for IC in Japan. Further advancements in treatment, including novel agent combinations and genomic profile-guided personalized medicine, are warranted to improve patient outcomes.

## Data Availability

The datasets generated and/or analyzed in the current study are available from the corresponding author upon reasonable request.
